# Evaluating the Role of Dietary Corn Particle Size on Production Traits and Egg Characteristics in Early‐Phase Laying Hens

**DOI:** 10.1002/fsn3.72247

**Published:** 2026-08-18

**Authors:** Kanon Das, Joohyun Ha, In Ho Kim

**Affiliations:** ^1^ Department of Animal Biotechnology Dankook University Cheonan Republic of Korea; ^2^ Smart Animal Bio Institute Dankook University Cheonan Republic of Korea

**Keywords:** diet, egg quality, fecal score, laying hen, nutrient, particle size

## Abstract

This study investigated the impact of corn particle size on laying hen performance, egg quality, egg size, and fecal score. A total of 252 Hy‐Line brown laying hens, aged 12 weeks, were randomly allotted to three dietary treatments with seven replicates of 12 hens per treatment. The experimental diets included corn particle sizes of 600 μm (TRT1), 900 μm (TRT2), and 1200 μm (TRT3) and were provided over an 18‐week trial. Results indicated that dietary corn particle size did not significantly affect egg production, feed conversion ratio (FCR), egg weight, egg size, or fecal score throughout the experimental period (*p* > 0.05). No significant effects were observed during weeks 12–18 and 18–24 for production performance or egg quality traits. During the late phase (24–30 weeks), average daily feed intake (ADFI) exhibited a quadratic tendency (*p* < 0.10), with reduced intake at the intermediate particle size (900 μm). Most egg quality parameters, including eggshell thickness, strength, Haugh unit, and albumen height, were not significantly influenced by dietary treatments (*p* > 0.05). However, yolk color showed a significant quadratic effect (*p* < 0.05), with lower values in hens fed 900 μm compared to 600 and 1200 μm diets, while eggshell color showed a tendency for a linear response (*p* < 0.10). These findings suggest that increasing corn particle size from 600 to 1200 μm maintains production performance and egg quality with minor phase‐specific effects, indicating that the inclusion of 1200 μm corn may be a practical strategy for laying hens.

## Graphical abstract

Feeding 600, 900, or 1200 μm corn particle sizes to early‐phase laying hens maintained stable production performance, egg quality, egg size, and fecal scores. Although average daily feed intake and yolk color exhibited a quadratic response from 24 to 30 weeks, the findings confirm that 1200 μm coarse corn is practically suitable for dietary inclusion without compromising overall productivity.

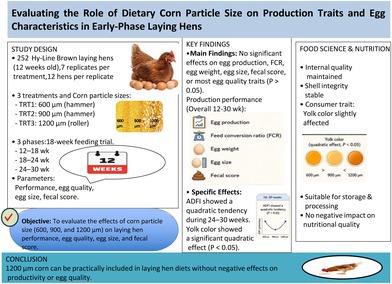

## 1 Introduction

The physical characteristics of poultry feed, particularly particle size, are significant determinants of nutrient utilization, gastrointestinal health, and overall production performance (Amerah et al. 2007; Nir et al. 1995). In laying hens, feed structure influences gizzard development, digesta retention time, and nutrient absorption efficiency, which all affect egg production and quality (Svihus 2011). Corn, a primary energy source in poultry diets, is frequently ground into different sizes to determine a balance between operational costs and ideal digestion. However, the relationship between corn particle size and laying performance remains contentious, with studies reporting conflicting outcomes depending on bird age, diet composition, and grinding method (Abdollahi et al. 2013).

Although the chemical composition of corn is constant, variations in particle size significantly affect the diet's nutritional value by altering nutrient digestibility and gastrointestinal physiology (Mtei et al. 2019). In addition to providing bioactive compounds with health‐promoting properties, yellow corn contains a complex profile of carotenoids, including xanthophylls (lutein, zeaxanthin, α‐ and β‐cryptoxanthin) and carotenes (α‐ and β‐carotene), which not only contribute to yolk pigmentation but also exert significant antioxidant, anti‐inflammatory, antibacterial, and immunomodulatory activities, thereby enhancing both avian physiological status and the nutritional quality of eggs for human consumption (Zurak et al. 2025). Fine corn particles generally improve starch gelatinization and enzymatic accessibility, whereas coarse particles stimulate gizzard development, prolong digesta retention time, and facilitate mechanical digestion (Röhe et al. 2014; Zaefarian et al. 2016). These effects may enhance digestive function and calcium availability for eggshell formation, which are essential for laying hens to influence eggshell weight and thickness in laying hens (Liu et al. 2023). In general, finer particles (< 600 μm) are associated with enhanced starch digestibility, whereas coarser particles (> 900 μm) are hypothesized to stimulate gizzard function, improve gut motility, and slow feed passage rate, potentially enhancing nutrient utilization and eggshell mineralization (Chewning et al. 2012; Engberg et al. 2002). Despite these proposed mechanisms, results from previous studies remain conflicting. For instance, Pérez‐Bonilla et al. (2011) observed improved feed efficiency in early lay cycles with coarser diets, while Karunajeewa and Tham (1984) reported minimal effects on egg weight and shell quality over longer periods.

Previous research on particle size effects in layers has predominantly focused on short‐term (starting at 06.00 AM and ending at 14.00 PM) performance metrics or specific egg quality parameters, with limited emphasis on continuous changes across production phases (Herrera et al. 2018). A phased analytical technique may be essential to identify temporal differences in laying hen responses to feed particle size. In addition to performance, egg quality traits such as Haugh unit, albumen height, and yolk pigmentation are commercially important, yet their relationship with particle size remains underexplored (Bain et al. 2016; Silversides et al. 2006). While particle size is known to influence calcium retention and shell calcification (Saki et al. 2019), its long‐term effects on dynamic parameters like eggshell color and albumen height remain poorly understood. For example, Lesson and Summers (2005) reported that larger particles slow digesta passage, allowing sustained nutrient absorption for yolk pigmentation, but factual evidence is limited. Similarly, Koçer et al. (2016) found improved shell thickness with coarse diets during early laying, but the benefits were not sustained throughout the production cycle.

Although prior research on corn particle size in laying hens has been conducted, most of these studies have concentrated on a particular production phase or overall means, leaving the temporal pattern of response during early lay inadequately defined. Therefore, by assessing three particle (600, 900, and 1200 μm) sizes over different early‐phase intervals, the current study expands on earlier research by determining whether dietary effects appear immediately after onset of lay or after physiological adaptation.

We assumed that (1) coarser corn particles would enhance early‐phase performance indicators such as feed intake and egg production due to improved intestinal function, and (2) egg quality parameters, particularly eggshell color and yolk color, would show particle size‐dependent responses, diminishing over time as hens adapt physiologically. Therefore, the purpose of this study is to observe early laying hens' performance, egg quality, egg size, and fecal score with different corn particle sizes.

## 2 Materials and Methods

The Animal Care and Use Committee of Dankook University in Cheonan, Republic of Korea, approved the research protocol (DK‐1‐2338) for this study.

### 2.1 Birds, Husbandry, and Experimental Diets

A total of 252 laying hens of the Hy‐Line brown breed, aged 12 weeks, were randomly distributed into three dietary treatment groups, each comprising seven replicates with twelve hens. The 18‐week trial took place in individual pens measuring 38 cm (width) × 50 cm (length) × 40 cm (height). The facility maintained a temperature of 26°C and the relative humidity controlled at approximately 60%, following a lighting schedule of 16 h of light and 8 h of darkness. Nipple drinkers and removable trough feeders were provided in each pen, ensuring unrestricted access to feed and water. The dietary groups included: (1) TRT1: 600 μm; (2) TRT2: 900 μm; and (3) TRT3: 1200 μm. Corn was processed using a hammer mill for 600 and 900 μm particles and a roller mill for 1200 μm particles. Corn was milled in a hammer mill equipped with a 3‐mm and a 10‐mm hammer mill screen and rolled in a roller with a gap of clearance of 1.8 mm and passed through a 10‐mm sieve opening. The particle size distribution of each feed was analyzed using a Ro‐Tap sieve shaker (Model RX‐29, W.S. Tyler, Mentor, OH, USA) equipped with a standard set of sieves (4.75–0.25 mm). The geometric mean particle size, geometric standard deviation of particle size, surface area and number of particles corn were calculated according to the ASAE S319.4 method. The experimental basal diet for laying hens, prepared and formulated in accordance with National Research Council, NRC (1994) standards Table 1, which outlines its ingredients and nutrient composition, with 16.50% crude protein (CP), 2800 kcal of metabolizable energy (ME)/kg, 4.08% Ca, 0.68% P, 0.88%, lysine, 0.79% methionine + cystine, and 0.45% of available phosphorus and the Korean Feeding Standard for Poultry (National Institute of Animal 2017).

**TABLE 1:** Ingredient composition and nutrient levels of the basal diet (as‐fed basis).

Item	Basal diet
Ingredients (%)	
Corn (8.8% CP)	61.39
Soybean meal (46.5% CP)	16.39
Corn gluten meal (60% CP)	4.94
Wheat bran (14.50% CP)	3.60
Tallow	0.97
MDCP	1.65
Limestone	9.75
Salt	0.25
Lysine (80%)	0.30
Methionine (50%)	0.26
Vitamin mix^a^	0.20
Mineral mix^b^	0.20
Choline (50%)	0.10
Total	100.00
Calculated value	
ME, kcal/kg	2800
Crude Protein, %	16.50
Crude Fat, %	3.43
Crude Fiber, %	2.89
Crude Ash, %	4.57
Calcium, %	4.08
Phosphorus, %	0.68
Available Phosphorus, %	0.45
Lysine, %	0.88
Methionine, %	0.45
Methionine + Cystine, %	0.79

^a^ Provided per kg of diet: vitamin A, 8000 IU; vitamin D3, 3300 IU; vitamin E, 20 g; vitamin K3, 2.5 g; vitamin B1, 2.5 g; vitamin B2, 5.5 g; vitamin B6, 4 g; vitamin B12, 23 mg; biotin, 75 mg; folic acid, 0.9 g; niacin, 30 g; D‐calcium pantothenate, 8 g.

^b^ Provided per kg of diet: Fe, 40 g as ferrous sulfate; Cu, 8 g as copper sulfate; Mn, 90 g as manganese oxide; Zn, 80 g as zinc oxide; 1.2 g as potassium iodide; and Se, 0.22 g as sodium selenite.

### 2.2 Sampling and Laboratory Analysis

The performance of laying hens was evaluated at 12–18, 18–24, and 24–30 weeks by monitoring parameters such as egg production, egg weight, feed intake, and FCR. Daily records of egg production, egg weights, and feed intake were maintained for each replication, and production percentage, FCR, and egg weight were calculated based on the recorded data for each replication. FCR was measured weekly and calculated by dividing total weekly feed intake by total egg mass.

Egg quality evaluations were carried out at the conclusion of the 12–18, 18–24, and 24–30 weeks of experimental duration. For the assessment of egg quality, a total of 210 eggs (70 eggs per treatment and 10 eggs per replicate), devoid of shell defects and cracks, were chosen at 17:00 h. Subsequently, on the same day at 20:00 h, measurements for egg quality were conducted.

Egg weight was determined using an egg multi tester (Touhoku Rhythm Co. Ltd., Tokyo, Japan). Eggshell breaking strength was measured with an eggshell force gauge (Model II, Robotmation Co. Ltd., Tokyo, Japan). Eggshell thickness was recorded using a dial pipe gauge (Ozaki MFG. Co. Ltd., Tokyo, Japan), based on the average thickness of the rounded end, pointed end, and equatorial region of the egg, excluding the inner membrane. Yolk color, albumen height, and Haugh unit (HU) were analyzed with an egg multi‐tester (Touhoku Rhythm Co. Ltd., Tokyo, Japan). Eggshell color was evaluated using an eggshell color fan (Dacho Ltd., Hwaseong, Korea).

Fresh excreta samples were collected from the 12–18, 18–24, and 24–30 weeks of experimental duration. The determination of fecal scores was done in accordance with the fecal quality scores (Lee et al. 2023). The fecal score varied from 1 to 5, with Score 1 representing dry and firm feces with characteristic white uric acid cover, Score 2 representing mostly dry feces with white uric acid cover, Score 3 representing moist feces with white uric acid cover, Score 4 representing wet feces with less white uric acid cover and droppings losing their shape, and Score 5 for extremely wet feces with little to no white uric acid cover. Scores were recorded based on observations of the overall stool consistency, and white paper was put on the plate after the removal of previous feces. The average fecal score was calculated by adding up the average daily fecal scores of each pen.

### 2.3 Statistical Analysis

All data were analyzed using the general linear model procedure of SAS (2013) in a randomized complete block design using pen as the experimental unit. Linear and quadratic polynomial contrasts were performed to determine the effects of the different treatments, and the means were separated using Tukey's multiple range test. Data variability was measured using the standard error of the mean (SEM). Differences were considered statistically significant at *p* < 0.05, whereas *p* < 0.10 was considered a tendency.

## 3 Results

Dietary Supplementation with different corn particle sizes (600, 900, and 1200 μm) did not significantly influence production performance, egg size, and fecal score in laying hens throughout the experimental periods. As shown in Table 2, from 12 to 18 weeks and 18 to 24 weeks, no linear or quadratic effects were observed on downgraded egg percentage, egg production, FCR, ADFI, and egg weight (*p* > 0.05). Similarly, during 24 to 30 weeks, these parameters remained unaffected, although ADFI showed a tendency for a quadratic effect (*p* < 0.10), with lower feed intake at the intermediate particle size (900 μm). Additionally, Table 3 presents egg quality traits, including eggshell color, Haugh unit, yolk color, albumen height, eggshell strength, and eggshell thickness, were not influenced during the early and mid‐phase (*p* > 0.05). However, during the late phase (24–30 weeks), a linear trend was observed for eggshell color (*p* < 0.10), while yolk color exhibited a significant quadratic effect (*p* < 0.05). Specifically, yolk color was lower in the 900 μm groups compared to the 600 and 1200 μm groups. Egg size was not affected by treatments at any stage (*p* > 0.05) (Table 4), fecal scores remained consistent across all groups throughout the study (*p* > 0.05) (Table 5). Overall, increasing corn particle size from 600 μm to 1200 μm maintained stable production and fecal consistency, with only minor, phase‐specific effects in yolk pigmentation and feed intake.

**TABLE 2:** The effects of particle size of corn feed on production performance in laying hens^a^.

Items	TRT1	TRT2	TRT3	SEM^b^	*p*	
					Linear	Quadratic
12–18 Week						
Downgraded egg, %	0.9	0.7	0.9	0.53	1.000	0.795
Egg production, %	7.5	7.9	8.1	0.59	0.507	0.985
FCR	36.23	31.64	29.4	3.61	0.196	0.792
ADFI, g	76	76	76	0.46	0.948	0.951
Egg weight, g	44.6	45.2	45.6	0.81	0.430	0.972
18–24 Week						
Downgraded egg, %	0.7	0.8	0.6	0.22	0.857	0.569
Egg production, %	75.3	75.1	75.2	0.71	0.933	0.885
FCR	2.92	2.9	2.92	0.07	1.000	0.772
ADFI, g	99.5	98.9	99.2	0.24	0.479	0.152
Egg weight, g	53.4	53.1	53.3	0.31	0.704	0.512
24–30 Week						
Downgraded egg, %	0.7	0.9	0.9	0.35	0.819	0.742
Egg production, %	95.7	95	95.4	0.36	0.471	0.254
FCR	1.94	1.96	1.95	0.02	0.728	0.688
ADFI, g	110.2	109.3	109.8	0.27	0.305	0.055**
Egg weight, g	59.6	58.9	59.2	0.63	0.684	0.505

^a^ TRT1, Control, Corn particle size (600 μm); TRT2, Corn particle size (900 μm); TRT3, Corn particle size (1200 μm).

^b^ Standard error of means.

** Indicates significance at *p* < 0.1.

**TABLE 3:** The effects of particle size corn feed on egg quality in laying hens^a^.

Items	TRT1	TRT2	TRT3	SEM^b^	*p*	
					Linear	Quadratic
12–18 Week						
Eggshell color	10.4	10.3	10.1	0.69	0.840	0.726
HU	94.9	95.1	94.7	2.05	0.946	0.991
Yolk color	8.5	8.3	8.8	0.37	0.573	0.332
Albumen height, mm	8.4	8.5	8.7	0.22	0.108	0.153
Eggshell strength, kg/cm^2^	4.6	4.55	4.57	0.13	0.892	0.777
Eggshell thickness, (×10^−2^ mm)	38.4	38.6	38.1	1.52	0.783	0.958
18–24 Week						
Eggshell color	10.4	10.3	10.3	0.26	0.426	0.712
HU	94.7	94.8	94.7	0.66	0.562	0.765
Yolk color	8.5	8.3	8.5	0.15	0.924	1.000
Albumen height, mm	8.2	8	8.2	0.16	0.600	0.271
Eggshell strength, kg/cm^2^	4.59	4.58	4.58	0.07	0.144	0.825
Eggshell thickness, (×10^−2^ mm)	38.3	38.4	38.3	0.56	0.531	0.281
24–30 Week						
Eggshell color	10.3	10.2	10.3	0.3	0.073**	0.426
HU	94.3	94.3	94.3	0.72	0.739	0.744
Yolk color	8.5	8.3	8.6	0.11	0.316	0.018*
Albumen height, mm	8.1	8.1	8.2	0.81	0.996	0.117
Eggshell strength, kg/cm^2^	4.59	4.56	4.58	0.05	0.261	0.524
Eggshell thickness, (×10^−2^ mm)	38.4	38.4	38.4	0.41	0.732	0.327

^a^ TRT1, Control, Corn particle size (600 μm); TRT2, Corn particle size (900 μm); TRT3, Corn particle size (1200 μm).

^b^ Standard error of means.

* Indicates significance at *p* < 0.05.

** Indicates significance at *p* < 0.1.

**TABLE 4:** The effects of particle size of corn feed on egg size in laying hens^a^.

Items	TRT1	TRT2	TRT3	SEM^b^	*p*	
					Linear	Quadratic
12–18 Week						
Less than 60 g	100	100	100	0	0	0
60 g or more	0	0	0	0	0	0
18–24 Week						
Less than 60 g	84.4	84.4	84.14	1.21	0.919	0.795
60 g or more	15.99	15.51	15.81	1.21	0.919	0.795
24–30 Week						
Less than 60 g	63.37	63.54	63.47	0.82	0.888	0.822
60 g or more	36.78	36.46	36.53	0.82	0.888	0.822

^a^ TRT1, Control, Corn particle size (600 μm); TRT2, Corn particle size (900 μm); TRT3, Corn particle size (1200 μm).

^b^ Standard error of means.

**TABLE 5:** The effects of particle size of corn feed on fecal score in laying hens^a^.

Items	TRT1	TRT2	TRT3	SEM^b^	*p*	
					Linear	Quadratic
Fecal score						
12–18 Week	1.6	1.6	1.6	0.05	0.867	0.387
18–24 Week	1.6	1.6	1.6	0.04	0.838	0.446
24–30 Week	1.6	1.7	1.6	0.05	0.614	0.515

^a^ TRT1, Control, Corn particle size (600 μm); TRT2, Corn particle size (900 μm); TRT3, Corn particle size (1200 μm).

^b^ Standard error of means.

## 4 Discussion

The present study evaluated the effects of different corn particle sizes on production performance, egg quality, egg size, and fecal characteristics in early‐phase laying hens from 12 to 30 weeks of age. The results revealed that increasing corn particle size did not adversely affect production performance, egg quality, egg size, or fecal consistency throughout the study, while only limited phase‐specific responses were observed for yolk pigmentation and feed intake during the late laying phase (24–30 weeks). These findings indicate that coarse corn particles can be incorporated into laying hen diets without compromising consumer‐relevant egg quality traits or product integrity, highlighting their relevance from both nutritional and food science perspectives. Especially, laying hens fed coarser corn particles showed minor phase‐specific responses in feed intake and egg pigmentation traits, particularly yolk color during the late laying phase. These findings provide significant observations into the influence of feed particle size on laying hen performance and egg quality during the early laying phase. Although no external feed additives were included in the present study, corn particle size functioned as a physical dietary modifier, influencing digestive dynamics and nutrient availability, which may indirectly affect feed efficiency, egg production, and shell quality (Yenice et al. 2025). These findings highlight that the physical characteristics of the diet can play a significant role similar to nutritional additives in optimizing laying hen productivity. Similarly, dietary additives such as essential oils, herbal powders, and seed‐derived products reported to improve laying hen performance and egg quality by enhancing antioxidant status and improving nutrient digestibility, indicating that both chemical and physical dietary modifiers can support egg production and shell and yolk traits (Elkomy et al. 2023; Saleh et al. 2021).

### 4.1 Effects on Production Performance

The results of this study suggest that dietary corn particle size had limited effects on production performance in laying hens throughout the experimental period. Egg production, egg weight, FCR, and ADFI were not significantly influenced by dietary treatments during all phases. Although hens fed the coarser corn diet (1200 μm) showed numerically higher egg production and egg weight, along with lower FCR during the early laying phase (12–18 weeks), these responses were not maintained during later phases. In addition, ADFI exhibited only a quadratic tendency during the late phase (24–30 weeks), with slightly lower feed intake observed in hens fed the intermediate particle size (900 μm). These findings are partially consistent with previous studies evaluating dietary particle size in laying hens. Oliveira et al. (2019) reported minimal effects of coarse corn particles on laying performance, although feed intake tended to increase in hens fed coarser diets. In contrast, Koçer et al. (2016) observed enhanced egg production and feed efficiency with larger corn particle sizes. Similarly, previous studies have suggested that larger particle sizes may enhance gastrointestinal function, leading to better nutrient utilization and improved performance in poultry (Amerah et al. 2007; Jacobs et al. 2010).

The limited and phase‐specific responses observed in the present study may be explained by differences in bird age, adaptation to diet structure, and the physiological transition into peak lay. The unusually high FCR values observed during the early phase were associated with very low egg production as hens entered the onset of lay.

Coarse particles may increase mechanical stimulation of the gizzard, leading to improved grinding activity, slower feed passage rate, and more efficient nutrient digestion and absorption. These physiological changes may improve energy utilization and protein availability, ultimately resulting in higher egg production, increased egg weight, and improved FCR. However, the present study did not evaluate gizzard development, nutrient digestibility, intestinal morphology, or retention time; therefore, the proposed mechanisms could not be directly confirmed. Further studies are needed to clarify the physiological effects of dietary corn particle size in laying hens. In addition, different grinding methods were used to produce experimental particle sizes, which may have influenced particle characteristics beyond size alone. Therefore, the present findings should be interpreted considering the potential confounding effects of feed processing methods. Overall, the findings indicate that increasing corn particle size from 600 to 1200 μm maintained stable long‐term laying performance without adverse effects on feed consumption or production efficiency.

### 4.2 Effects on Egg Quality

Eggshell thickness, eggshell strength, Haugh unit, and albumen height were not significantly influenced by dietary treatments, indicating that increasing corn particle size from 600 to 1200 μm did not adversely affect egg structural integrity or internal egg quality. However, during the late laying phase (24–30 weeks), yolk color exhibited a significant quadratic response, with lower pigmentation observed in hens fed the 900 μm diet compared with the 600 and 1200 μm treatments. In addition, eggshell color showed a linear tendency during the same phase. Overall, these findings suggest that dietary corn particle size had only minor phase‐specific effects on egg pigmentation while maintaining overall egg quality.

These findings are consistent with previous studies reporting minimal effects of feed particle size on internal egg quality traits. Ruan et al. (2018) reported no marked effects on Haugh unit, albumen height, or yolk color, whereas Svihus (2011) suggested that coarse particles may improve digestive activity and nutrient utilization. In the present study, pigmentation responses were observed only during the late laying phase, suggesting that the effects of dietary particle size on egg pigmentation may be gradual or cumulative, consistent with previous findings (Ojelade 2016).

The observed changes in yolk pigmentation may be associated with differences in nutrient digestion and carotenoid availability influenced by feed particle structure. Previous studies have also shown that dietary additives can improve egg quality and antioxidant status in laying hens (Xu et al. 2025), suggesting that physical dietary characteristics may similarly influence nutrient utilization. In the current study, corn particle size acted as a physical dietary modifier rather than a chemical additive, suggesting that feed structure itself may contribute to maintaining egg quality.

### 4.3 Effects on Egg Size

Our results showed stable egg size characteristics across treatments, suggesting that moderate differences in feed particle structure did not markedly influence nutrient partitioning associated with egg formation in laying hens, while indicating that coarse grinding can be applied without adversely affecting commercially important egg grading traits. These results are consistent with previous studies reporting limited effects of feed particle size on egg size and egg mass characteristics (Ege et al. 2019). In contrast, some previous studies have reported increased egg size in hens fed coarse particle diets, potentially due to improved nutrient utilization and digestive efficiency (Summers and Leeson 1983). Similarly, Zhang JiaQi et al. (2018) suggested that the effects of dietary structure on egg characteristics may decline as hens approach peak production. In the present study, the absence of significant treatment effects across all phases indicates that hens maintained stable egg size regardless of dietary particle size. A possible reason for the limited response is that moderate differences in corn particle size were insufficient to substantially alter nutrient availability associated with egg size formation. Although coarse particles may improve digestive activity and nutrient utilization, these effects were not sufficient to produce consistent changes in egg size in the present study.

### 4.4 Effects on Fecal Characteristics

Fecal scores were not significantly affected by the corn particle size in laying hens across all experimental periods (12–18, 18–24, and 24–30 weeks). All treatments TRT1, TRT2, and TRT3 resulted in comparable fecal scores, with no significant differences in excretions of raw excreta, dry excreta, and moisture among the dietary groups (Kolawole and Kim 2025). The present observations that corn particle size may not be a critical determinant of fecal consistency in laying hens.

## 5 Conclusion

Dietary corn particle size affected feed intake and yolk pigmentation during the peak laying phase (24–30 weeks), but it had limited effects on laying hen performance. Thus, we suggest that 1200 μm particle size would be beneficial for maintaining the yolk color and feed efficiency without compromising egg structural integrity.

## Author Contributions

**Joohyun Ha:** conceptualization, methodology, writing – original draft. **Kanon Das:** conceptualization, methodology, investigation, writing – original draft, validation, visualization, writing – review and editing, software, formal analysis. **In Ho Kim:** conceptualization, funding acquisition, investigation, project administration, writing – review and editing, supervision, resources, validation, methodology.

## Funding

This work was supported by the National Research Foundation of Korea (NRF) and the Ministry of Education (NRF‐RS‐2023‐00275307).

## Ethics Statement

The Animal Care and Use Committee of Dankook University gave its approval to all procedures. The procedures were developed to meet the standards of the European Union (2010/63/EU Directive).

## Conflicts of Interest

The authors declare no conflicts of interest.

## Data Availability

The data that support the findings of this study are available on request from the corresponding author. The data are not publicly available due to privacy or ethical restrictions.
